# Effectiveness and Safety of High-Dose Dual Therapy: Results of the European Registry on the Management of *Helicobacter* *pylori* Infection (Hp-EuReg)

**DOI:** 10.3390/jcm11123544

**Published:** 2022-06-20

**Authors:** Luis Fernández-Salazar, Ana Campillo, Luis Rodrigo, Ángeles Pérez-Aisa, Jesús M. González-Santiago, Xavier Segarra Ortega, Maja Denkovski, Natasa Brglez Jurecic, Luis Bujanda, Blas José Gómez Rodríguez, Juan Ortuño, Sotirios Georgopoulos, Laimas Jonaitis, Ignasi Puig, Olga P. Nyssen, Francis Megraud, Colm O’Morain, Javier P. Gisbert

**Affiliations:** 1 Hospital Clínico Universitario Valladolid, Gerencia Regional de Salud (SACYL), Facultad de Medicina, Universidad de Valladolid, 47002 Valladolid, Spain; 2 Hospital Reina Sofía, 31500 Tudela, Spain; campilloarregui@gmail.com; 3 Hospital Universitario Central de Asturias, 33011 Oviedo, Spain; lrodrigosaez@gmail.com; 4 Hospital Costa del Sol, Redes de Investigación Cooperativa Orientada a Resultados en Salud (RICORS), 29603 Marbella, Spain; drapereza@hotmail.com; 5 Complejo Asistencial Universitario de Salamanca, Centro de Investigación Biomédica en Red de Enfermedades Hepáticas y Digestivas (CIBERehd), Instituto de Investigación de Salamanca (IBSAL), 37007 Salamanca, Spain; jmgonzalezsantiago@gmail.com (J.M.G.-S.); mdjosexaviersegarra9@gmail.com (X.S.O.); 6 Interni Oddelek, Diagnostic Centre, 4260 Bled, Slovenia; maja.denkovski@gmail.com (M.D.); natasa.brglez.jurecic@gmail.com (N.B.J.); 7 Hospital Donostia, Instituto Biodonostia, Centro de Investigación Biomédica en Red de Enfermedades Hepáticas y Digestivas (CIBERehd), Universidad del País Vasco (UPV/EHU), 20018 San Sebastián, Spain; medik@telefonica.net; 8 Hospital Virgen de la Macarena, 41009 Sevilla, Spain; gomezblasj@gmail.com; 9 Hospital Universitari i Politècnic, La Fe, 46026 Valencia, Spain; jortunoc@comv.es; 10 Athens Medical Centre, Paleo Faliron Hospital, 17562 Athens, Greece; georgpap@ath.forthnet.gr; 11 Department of Gastroenterology, Institute for Digestive Research, Lithuanian University of Health Sciences, 44307 Kaunas, Lithuania; laimasjonaitis@yahoo.com; 12 Gastroenterology Service, Althaia Xarxa Assistencial Universitària de Manresa, 082443 Manresa, Spain; ignasi.puig@aegastro.es; 13 Medicine Department, Universitat de Vic-Universitat Central de Catalunya (UVicUCC), 08500 Vic, Spain; 14 Centro de Investigación Biomédica en Red de Enfermedades Hepáticas y Digestivas (CIBEREHD), Hospital Universitario de La Princesa, Instituto de Investigación Sanitaria Princesa (IIS-Princesa), Universidad Autónoma de Madrid (UAM), 28006 Madrid, Spain; opn.aegredcap@aegastro.es (O.P.N.); javier.p.gisbert@gmail.com (J.P.G.); 15 INSERM U1312, Université de Bordeaux, 33000 Bordeaux, France; francis.megraud@u-bordeaux.fr; 16 Faculty of Health Science, Trinity College Dublin, 02 PN40 Dublin, Ireland; colmomorain@gmail.com

**Keywords:** *Helicobacter pylori*, therapy, amoxicillin, registry, high-dose, eradication, effectiveness, rescue treatment

## Abstract

Background: Randomized clinical trials and meta-analyses, primarily from Asian countries, have reported good effectiveness with high-dose dual therapy (HDDT) including a proton pump inhibitor (PPI) and amoxicillin when prescribed as *H. pylori* first-line or rescue treatment. However, combining amoxicillin with PPIs in the 1990s in several European countries yielded suboptimal results. Methods: An international, multicenter, prospective non-interventional Registry (Hp-EuReg) aimed to evaluate the decisions and outcomes of *H. pylori* management by European gastroenterologists. All infected adult cases treated with HDDT were registered at e-CRF AEG-REDCap platform until June 2021. Sixty patients were prescribed with HDDT (98% compliance), 19 of them received a first-line therapy and 41 a rescue treatment (second- to sixth-line). Results: Overall HDDT effectiveness was 52% (per-protocol) and 51% (modified intention-to-treat). First-line and rescue treatment lines were equally effective, but the effectiveness was worse when patients had previously received metronidazole, tetracycline, or rifabutin. Adding bismuth to HDDT in rescue treatment did not yield better results. The incidence of adverse events was 30%, diarrhea being the most common (20% of patients); no serious adverse events were reported. Conclusion: Although HDDT is safe and has good compliance, it is not a good option in European first-line or rescue *H. pylori* treatment, even when adding bismuth.

## 1. Introduction

*Helicobacter pylori* infection has a worldwide prevalence of 50% and is the main cause of chronic gastritis, peptic ulcer, and gastric cancer, diseases of great clinical and socio-economic importance; there is an ongoing debate over its management [1]. There are numerous different regimens that vary in both antibiotic type and proton pump inhibitor (PPI) dose, as well as the duration of *H. pylori* treatment. For instance, the European Registry on *Helicobacter pylori* Management (Hp-EuReg) includes more than 100 different first-line treatment schemes [2]. Since 1997, the triple therapy based on a PPI plus amoxicillin and clarithromycin or metronidazole has been the treatment recommended in first-line infection [3]. It was formerly the most commonly used [2] given its initial higher effectiveness as compared to the dual association of amoxicillin and a PPI [4,5,6]. However, the ideal goal should be to cure the infection with a success rate of at least 90%, and such triple therapy fails to eradicate the bacteria in at least 20–30% of cases [2]. A major reason for treatment failure is acquired bacterial antibiotic resistance (mainly to clarithromycin), which has been gradually increasing worldwide [7].

Quadruple regimens (mainly bismuth-containing quadruple therapies) are the recommended first-line therapies where resistance to clarithromycin is over 15% [1], which is currently the case in most European countries [7]. However, even after treatment with these quadruple regimens, a considerable number of patients have persistent *H. pylori* infection. In contrast, several randomized clinical trials (RCTs) and meta-analyses, mostly from Asian countries, have reported optimal efficacy and safety rates with high doses of amoxicillin and a PPI—that is, high-dose dual therapy (HDDT)—when prescribed either as first-line or as a rescue treatment [8,9,10,11,12]. These studies demonstrate that HDDT efficacy in Asian countries is equivalent, or even superior, to different recommended therapies, such as concomitant quadruple therapy, or therapies including levofloxacin, bismuth, or rifabutin [8,9,10,11,12].

In the current study, we aimed to evaluate the frequency of use, the effectiveness, the compliance, and the safety of the HDDT regimen in the management of *H. pylori* infection in Europe, where resistance rates and genetic and environmental aspects could yield different results from those found in Asian countries.

## 2. Methods

### 2.1. European Registry on H. pylori Management

The Hp-EuReg is an international multicenter prospective non-interventional registry founded in 2013 and promoted by the European Helicobacter and Microbiota Study Group (www.helicobacter.org) that started in 2013. The Hp-EuReg protocol was approved by the Ethics Committee of La Princesa University Hospital (Madrid, Spain) [13] and was registered at ClinicalTrials.gov with code NCT02328131. The study protocol conforms to the ethical guidelines of the 1975 Declaration of Helsinki as reflected in a prior approval by the institution’s human research committee.

Country selection criteria, national coordinators, and further details on the variables collected (including demographics, comorbidity, data on infection and diagnosis, previous eradication attempts, current treatment, compliance, adverse events (AEs) and effectiveness) are reported in the published protocol [13]. Participating investigators were gastroenterologists who routinely managed patients in whom *H. pylori* eradication treatment was indicated.

### 2.2. Data Collection

Data were collected in an electronic case report form (e-CRF), using the collaborative platform Research Electronic Data Capture (REDCap) hosted at “Asociación Española de Gastroenterología” (AEG; www.aegastro.es) [14]. All personal data were anonymized. Written, informed consent was obtained from each patient included in the study. All patients registered until June 2021 were included in the present analysis.

### 2.3. Study Aim

The objective of the current analysis was to evaluate the frequency of use, effectiveness, compliance, and safety of HDDT in the management of *H. pylori* infection in first-line treatment and as a rescue therapy (second to sixth line of treatment), and also when HDDT was prescribed in combination with bismuth.

### 2.4. Selection Criteria

Patients receiving HDDT (amoxicillin 1000 mg three times a day plus a PPI), with or without bismuth, in any treatment line and for at least 10 days, were selected for inclusion.

### 2.5. Data Management

Continuous variables were summarized using the mean and standard deviation (SD). Qualitative variables were summarized using absolute and relative frequencies with percentages (%), and 95% confidence intervals (95% CI) were provided. Differences between groups were analyzed with the Chi-square test. Statistical significance was set as *p* < 0.05.

PPI doses were categorized into three groups according to the potency of acid inhibition: low-dose (4.5–27 mg of omeprazole equivalents given twice a day), standard-dose (32–40 mg of omeprazole equivalents given twice a day), or high-dose (54–128 mg of omeprazole equivalents given twice a day) [15,16]. The duration of treatment was categorized as 7, 10, or 14 days, to facilitate data interpretation.

### 2.6. Effectiveness Analysis

Treatment eradication rate was the main outcome and had to be confirmed at least four weeks after treatment using locally accepted and validated diagnostic methods. Effectiveness was studied in three sets of patients. First, an intention-to-treat (ITT) group included all patients registered up to June 2021, allowing at least a 6-month follow-up, and considering patients lost to follow-up as treatment failures. The per-protocol (PP) set included all compliant patients (i.e., who had taken ≥90% of the prescribed drugs) who had completed follow-up. A modified ITT (mITT) group was defined to reflect the result closest to those obtained in the clinical practice and included all patients who had completed follow-up (i.e., a confirmatory test—success or failure—was available after eradication treatment), regardless of compliance.

### 2.7. Safety and Compliance

AEs and compliance were evaluated through patient questioning using both open-ended questions and a predefined questionnaire. Compliance was defined as having taken ≥90% of the prescribed drugs. AEs were classified depending on the intensity of symptoms evaluated by the corresponding physician: mild (not interfering with daily routine), moderate (affecting daily activities), intense/severe (not allowing normal daily activities), and serious (causing death, hospitalization, disability, congenital anomaly, and/or requiring intervention to prevent permanent damage).

## 3. Results

### 3.1. Baseline Characteristics

Sixty patients out of 44,504 included in the Hp-EuReg received HDDT. Thirty-nine were women (65%) and the mean age was 42 years (SD 15). Fifty-five of them (92%) were registered in Spain, two in Slovenia and one each in France, Greece and Lithuania. The indication for treatment was uninvestigated dyspepsia in 22 cases (37%), functional dyspepsia in 20 (33%), and duodenal ulcer in seven (12%), with 11 other indications (such as a family history of gastric cancer) in the remaining patients.

### 3.2. Treatment Lines

Nineteen (32%) out of 60 patients received HDDT as first-line treatment. The remaining 41 patients (68%) received the therapy as rescue treatment; that is, they had previously received at least one prior treatment. Of those 41 non-naive patients, four patients received HDDT as second line, five as third line, two as fourth line, 23 as fifth line, and seven as sixth line. The total number of different treatments prior to HDDT in those 41 non-naive patients was 147 and is presented in the Supplementary Table S1. The different antibiotics prescribed to the 60 patients as first- or rescue lines are presented in Table 1.

### 3.3. Prescriptions

All 60 patients received 1000 mg of amoxicillin three times a day. The most frequently used PPI was esomeprazole 40 mg in 56 patients (93%); esomeprazole was prescribed three times a day in 52 patients, and twice a day in four. Two patients were treated with omeprazole, one of them with 40 mg three times a day and the other with 20 mg three times a day. One patient received pantoprazole 40 mg three times a day, and the last one received 20 mg of rabeprazole three times a day. Acid suppression potency was classified as high in 53 patients (93%), standard in two patients (3.3%), and low in another two patients (3.3%). The duration of treatment was 14 days in 58 cases (97%) and 10 days in two (3.3%). All the treatment-naïve patients were prescribed high potency of acid inhibition for 14 days; while 35 patients among the 41 cases receiving a rescue treatment were prescribed with high-doses of PPIs and 14 days of therapy. Bismuth was added to HDDT in 13 cases (22%). The dose was 120 mg twice a day in 11 of them, and 240 mg twice a day in another two.

### 3.4. HDDT Effectiveness

**Figure 1:**
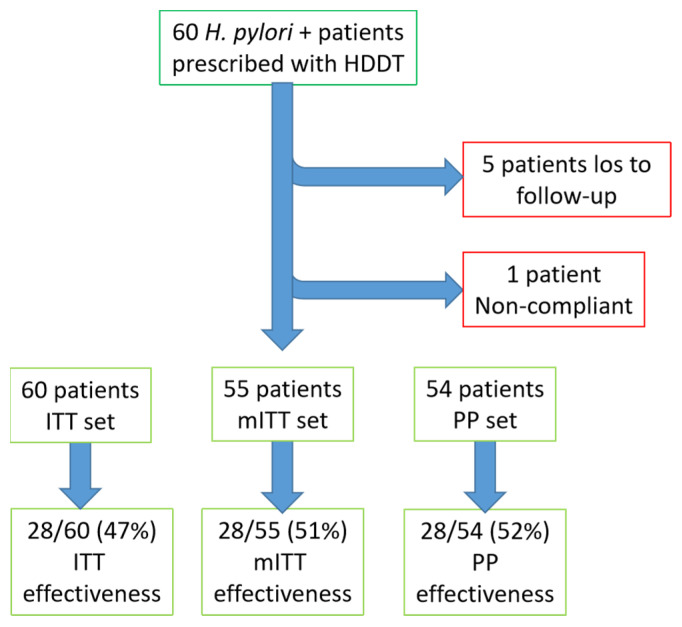
Study flowchart. HDDT, high-dose dual therapy; ITT, intention to treat; mITT, modified intention to treat; PP, per protocol.

HDDT success or failure was determined with ^13^C-urea breath test in 55 patients. The overall mITT eradication rate of HDDT was 51% (28/55) CI 37–64. The study flowchart is shown in Figure 1.

There were no statistical differences between treatment lines, either between naive vs. non-naive patients (*p* > 0.05) (Table 2). The effectiveness of HDDT was below 70% in all scenarios (both first-line and rescue treatment). Among the 35 patients treated with the optimized rescue HDDT therapy (that is, high-dose PPIs for 14 days), the effectiveness was similar (mITT 46.9%; 15/32) to the overall rescue (encompassing all treatment schemes) treatment effectiveness (mITT 44.7%, 17/38).

Patients who had previously received metronidazole or tinidazole, tetracycline or doxycycline, or rifabutin had a significantly worse HDDT eradication rate (*p* < 0.001) (Table 3).

### 3.5. Effectiveness of HDDT + Bismuth

Bismuth was added to HDDT in 13 patients: four patients with five previous treatments, eight with four previous treatments, and one patient with one previous treatment. The results did not show a significantly higher overall eradication rate compared to HDDT alone (Table 4).

### 3.6. Compliance and Safety

One patient (1/55, 2%), who had received four previous regimens, did not comply with treatment. All remaining cases reported good adherence to treatment.

There were 17 cases (17/55, 31%) reporting at least one AE (Table 5). They were all of mild or moderate intensity, except for one case of anorexia that was considered severe. The AEs lasted up to 7 days, except for three cases of anorexia, one of asthenia, one of abdominal pain, and another of dyspepsia; these all lasted for 11 days. No serious AEs were reported.

## 4. Discussion

Given its great tolerance, the treatment combination of different amoxicillin dosages plus a PPI was used in the 1990s, achieving highly variable cure rates that ranged from 50% to more than 80% [17,18]. In the Hp-EuReg, HDDT effectiveness in naive patients was less than 65% by mITT and PP analyses, which was far below the desired 90% threshold. As a first-line treatment, HDDT effectiveness was lower than the one obtained with quadruple regimens with or without bismuth; its effectiveness was also lower when bismuth was added to the triple regimen, which reached 90% cure rates in European countries [2]. As a rescue treatment, overall HDDT effectiveness was 45% by mITT analyses in the Hp-EuReg, and this rate was even lower in patients who had previously received metronidazole (32%), tetracycline (26%) or rifabutin (25%).

However, the data on HDDT effectiveness and efficacy are much better in different RCTs and several recent meta-analyses from Asian countries. These studies find cure rates similar to or even higher than those of generally recommended therapies [8,9,10,11,12,19,20]. Some possible explanations for these good results are:

- The lower amoxicillin resistance rate of *H. pylori* compared to other antibiotics [21].
- The advantages of achieving and maintaining high plasma concentrations of amoxicillin thanks to the sequential administration of high doses of amoxicillin three or four times a day; some studies suggest that administering 750 mg of amoxicillin four times a day is superior to administering 1 g three times a day [10] and consider this regimen better than the standard triple, the standard triple with bismuth or the classic bismuth quadruple therapies [11].
- The powerful and persistent suppression of gastric acid secretion with the frequent administration of high PPI doses, and increasing *H. pylori* sensitivity to amoxicillin [22].
- The differences in cytochrome P450 CYP2C19 polymorphisms between the European and Asian populations could also influence the differences in HDDT effectiveness depending on the PPI doses and types [23,24].
- Treatment duration, with 14-day prescriptions, is also considered a relevant factor to ensure HDDT effectiveness [11,25,26], as is the case with other treatments [2].

One of the above-mentioned meta-analyses from 2019 (four RCTs and 829 patients) reported an overall HDDT efficacy equivalent to bismuth therapies (ITT 85.5% vs. 87.2%) [8]. Another 2019 meta-analysis compared the HDDT regimen vs. all-in-one group therapies (standard triple therapy with and without bismuth, concomitant quadruple regimen, bismuth quadruple regimen, and rifabutin triple therapy), also showing similar efficacy. Two sub-analyses demonstrated similar HDDT efficacy in naive patients and in patients receiving a second-line treatment [9]. A recent meta-analysis (15 RCTs and 3818 patients) also reported overall HDDT efficacy comparable to the one reported in an all-in-one group of different recommended therapies (standard triple therapy with and without bismuth, levofloxacin triple therapy, bismuth quadruple therapy, and sequential regimen and rifabutin triple therapy) [10]. A more recent meta-analysis comparing HDDT with several regimens recommended as first-line treatment (HDDT vs. standard triple therapy, bismuth triple or quadruple therapy, and non-bismuth quadruple therapy) did not reveal significant differences between groups in overall eradication rate. Additionally, sub-analyses of two studies including only treatments without clarithromycin, and three studies reporting antibiotic susceptibility test results, did not show significant differences either [12]. Lastly, the superiority of HDDT over the standard triple therapy, triple therapy with bismuth, and classic bismuth quadruple regimen has been reported in a very recent meta-analysis. HDDT efficacy was higher as a first-line therapy (89.8% vs. 84.2%) and also in the total of the patients receiving first-line and rescue treatment (89.7% vs. 84.6%) [11].

Two network meta-analyses also explored the eradication rates of different first-line treatment regimens [19,27]. One of them included 41 RCTs comparing mainly concomitant and sequential therapy but also reverse hybrid therapy, as well as different bismuth-based quadruple therapies, where five studies included a HDDT arm. The results of this meta-analysis suggested that 14-day HDDT was the preferred first-line treatment in the Asian population [27]. Another network meta-analysis (68 studies, three of them with an HDDT arm) showed that HDDT was more effective than bismuth-based triple and quadruple therapies but less effective than non-bismuth concomitant, sequential, and reverse hybrid quadruple treatments, levofloxacin-containing therapy, and vonoprazan-triple therapy [19].

Two meta-analyses showed higher HDDT effectiveness in studies in Asian countries than in European ones [8,9,10]. In addition, a sub-analysis in one network meta-analysis exploring the regional effect on cure rates placed HDDT last in Western countries [19]. In line with this, also within Europe differences in the effectiveness of the different regimens have been described between countries and distant geographical areas [2]. A 2015 multicenter Italian study showed a good 10-day HDDT efficacy of 87.5% [28], while a randomized trial in Latvia recently reported that HDDT plus bismuth had lower cure rates than standard triple therapy by PP analysis (77% vs. 88.4%) [29]. Ethnic and biological differences among different geographic areas could probably explain the discordant results between these meta-analyses and most of the experiences in European countries.

In Hp-EuReg, the AE incidence rate with HDDT was nearly 30%. Diarrhea, nausea, and asthenia were the most frequently reported events, most of them mild, short-lasting cases. HDDT treatment showed a lower AE rate than bismuth quadruple therapy (14.4% vs. 40%) [8], as well as a lower rate than different all-in-one groups of recommended regimens including standard triple therapy, standard triple therapy with bismuth, concomitant quadruple regimen, bismuth quadruple regimen, and rifabutin triple therapy [10,11,12]. The most frequent AEs described with HDDT in these studies are similar to the ones we report in our study: diarrhea, nausea, and dizziness [9,10,11], abdominal pain, and metallic taste [11], generally mild and of short duration.

Adherence to HDDT was higher than 90% in most (>95%) Hp-EuReg patients. This is a key factor that has been associated with higher cure rates [2,30]. However, despite the fact that HDDT shows better tolerance than other recommended therapies in the different studies, better compliance was not necessarily reported [8,9,11,29]. In fact, one of the network meta-analyses places HDDT in the worst place in terms of compliance compared to bismuth-based, concomitant, and sequential quadruple therapies. HDDT is last in compliance in both total patients and in the Asian population sub-analysis [27].

Our results and conclusions are based on data in the Hp-EuReg, a multicenter prospective registry that reflects routine clinical practice. The 60 patients included in our study, mostly registered in Spain, were treated with 3 g of amoxicillin per day divided into three doses, and most of them, plus a high-dose PPI regimen [15,16], also distributed in three doses for 14 days. This is a very different scenario from the RCTs and meta-analyses, which deal with a high number of patients and strict inclusion and exclusion criteria [8,9,10,19,27]. The main limitations of our study are the reduced number of patients and the inevitable variability in terms of the number and treatment regimens received before HDDT, reflecting clinical practice.

In conclusion, based on the Hp-EuReg results, HDDT seems to be a safe, well-tolerated option; however, its effectiveness in clinical practice and in our environment, both in naive patients and as rescue therapy, is far from the desirable threshold of effectiveness even when bismuth is added. Consequently, HDDT does not seem to represent a good treatment option in Europe.

## Figures and Tables

**Table 1:** Antibiotic usage before HDDT in non-naive patients.

	Rescue Patients n/N (%)
Amoxicillin	40/41 (97.5%)
Clarithromycin	38/41 (92.6%)
Levofloxacin/Moxifloxacin	35/41 (85.3%)
Bismuth salts	31/41 (75.6%)
Metronidazole/tinidazole	30/41 (73.1%)
Tetracycline/Doxycycline	24/41 (58.5%)
Rifabutin	20/41 (48.7%)
Rifaximin	4/41 (9.7%)

HDDT, high-dose dual therapy; n: number of patients receiving different antibiotics at least once before HDDT; N: non-naive patients.

**Table 2:** HDDT effectiveness in first-line and as rescue treatment.

	ITT n/N (%)	*p* *	PP n/N (%)	*p* *	mITT n/N (%)	*p* *
HDDT overall	28/60 (46.7%) CI 38–65		28/54 (51.9%) CI 38–65		28/55 (50.9%) CI 37–64	
HDDT 1st line **	11/19 (57.9%) CI 39–90	0.291	11/17 (64.7%) CI 39–90	0.278	11/17 (64.7%) CI 39–90	0.234
HDDT rescue treatment (from 2nd to 6th line) ***	15/35 (42.9%) CI 29–67		15/31 (48.4%) CI 29–67		15/32 (46.9%) CI 29–67	
HDDT rescue treatment (from 2nd to 6th line)	17/41 (41.5%) CI 29–62		17/37 (45.9%) CI 29–62		17/38 (44.7%) CI 29–62	
HDDT 2nd line	2/4 (50%)	0.187	2/3 (66.7%)	0.102	2/3 (66.7%)	0.110
HDDT 3rd line	2/5 (40%)		2/4 (50%)		2/4 (50%)	
HDDT 4th line	1/ 2 (50%)		1/ 2 (50%)		1/ 2 (50%)	
HDDT 5th line	12/23 (52.2%)		12/21 (57.1%)		12/22 (54.5%)	
HDDT 6th line	0/7 (0%)		0/7 (0%)		0/7 (0%)	

CI, 95% confidence interval; HDDT, high-dose dual therapy; ITT intention to treat; mITT modified intention-to-treat; n, number of patients with *H. pylori* infection cured; N, number of patients treated; PP per protocol. * There were no statistically significant differences in the HDDT eradication rate between first-line and all subsequent rescue treatment lines; neither were there statistically significant differences when the different rescue lines were compared. The Chi-square test was used at a significance level of *p* < 0.005. ** All treatment-naïve cases were treated with HDDT at high-doses PPIs for 14 days. *** Among the 41 rescue treatment cases, 35 were treated with HDDT at high-doses PPIs for 14 days.

**Table 3:** Comparison of HDDT effectiveness by previous antibiotic prescriptions.

	ITT n/N (%)		*p*	PP n/N (%)		*p*	mITT n/N (%)		*p*
	Not Previously Used	Previously Used		Not Previously Used	Previously Used		Not Previously Used	Previously Used	
A	11/20 (55%)	17/40 (42.5%)	0.360	11/17 (64.7%)	17/37 (45.9%)	0.200	11/17 (64.7%)	17/38 (45.9%)	0.171
C	12/22 (54.5%)	16/38 (42.1%)	0.352	12/19 (63.2%)	16/35 (45.7%)	0.221	12/19 (63.7%)	16/36 (44.4%)	0.187
M-T	19/30 (63.3%)	9/30 (30.1%)	0.010	19/27 (70.4%)	9/27 (33.3%)	0.006	19/27 (70.4%)	9/28 (32.1%)	0.005
L-Mx	13/25 (52%)	15/35 (42.9%	0.484	13/22 (59.1%)	15/32 (46.9%)	0.377	13/22 (59.1%)	15/33 (45.5%)	0.322
B	15/29 (51.7%)	13/31 (41.9%)	0.448	15/24 (60%)	13/29 (44.8%)	0.266	15/25 (60%)	13/30 (43.3%)	0.218
Tc-Dc	22/36 (61.1%)	6/24 (25%)	0.006	22/32 (68.8%)	6/22 (27.3%)	0.003	22/32 (68.8%)	6/23 (26.1%)	0.002
Rf	23/40 (57.5%)	5/20 (25%)	0.017	23/35 (65.7%)	5/19 (22.7%)	0.006	23/35(65.7%)	5/20 (25%)	0.004

A, amoxicillin; B, bismuth salts; C, clarithromycin; Conc, concomitant; HDDT, high-dose dual therapy; Hyb, hybrid; ITT, intention to treat; L-Mx, levofloxacin or moxifloxacin; M-T, metronidazole or tinidazole; mITT, modified intention-to-treat; PP, per protocol; PPI, proton pump inhibitor; Rf, rifabutin; Seq, sequential; Tc-Dc, tetracycline or doxycycline. HDDT eradication rates in patients previously treated with different antibiotics. The Chi-square test was used at a significance level of *p* < 0.005.

**Table 4:** Effectiveness of HDDT rescue treatment with and without bismuth.

	ITT n/N (%)	*p* *	PP n/N (%)	*p* *	mITT n/N (%)	*p* *
With B	4/13 (30.8%)	0.344	4/12 (33.3%)	0.286	4/13 (30.8%)	0.212
Without B	13/28 (46.4%)		13/25 (52%)		13/25 (52%)	

B, bismuth; HDDT, high-dose dual therapy; ITT, intention to treat; mITT, modified intention to treat; n: number of patients with *H. pylori* infection cured; N, number of patients treated; PP, per protocol. * Adding bismuth to HDDT showed no statistically significant association with a higher eradication rate in the rescue treatment. The Chi-square test was used at a significance level of *p* < 0.005.

**Table 5:** Safety with HDDT.

Adverse Events	n/N (%)
Diarrhea	11/55 (20%)
Nausea	6/55 (11%)
Asthenia	5/55 (11%)
Abdominal pain	4/55 (7%)
Anorexia	4/55 (7%)
Dyspepsia	3/55 (5%)
Dizziness	2/55 (3%)
Headache	2/55 (3%)
Vomits	2/55 (3%)
Heartburn	0/55 (0%)
Metallic taste	0/55 (0%)

HDDT, high-dose dual therapy; n, number of patients with at least one adverse event; N, number of patients reporting adverse events.

## Data Availability

The data that support the findings of this study are not publicly available given that containing information could compromise the privacy of research participants. However, previous published data on the Hp-EuReg study, or de-identified raw data referring to current study, as well as further information on the methods used to explore the data could be shared, with no particular time constraint. Individual participant data will not be shared.

## Supplementary Materials

The following supporting information can be downloaded at: https://www.mdpi.com/article/10.3390/jcm11123544/s1, Table S1: Regimens used as first and rescue treatment lines in the 60 patients who received HDDT.
