# Supplementary material for: Effectiveness and Safety of High-Dose Dual Therapy: Results of the European Registry on the Management of Helicobacter pylori Infection (Hp-EuReg)
Source: J Clin Med. 2022 Jun 20;11(12):3544. doi: 10.3390/jcm11123544 (PMC9225562; doi:10.3390/jcm11123544)
Supplement: Supplementary file 1 [file jcm-11-03544-s001.zip › jcm-1718158-supplementary.pdf]

Journal of
Clinical Medicine

MDPI

Supplementary file.

Table S1. Regimens used as first and rescue treatment lines in the 60 patients who received HDDT.

Treatment line	PPI-A(B) HDDT n/N (%)	PPI-M+Tc+B n/N (%)	PPI-C+A n/N (%)	PPI-A+L/M n/N (%)	PPI-A+Rf n/N (%)	PPI-C+A+M/T Conc n/N (%)	PPI+ single capsule* n/N (%)	PPI-A+L/M+B n/N (%)	PPI-A+M/T n/N (%)	PPI-A+Rf+B n/N (%)	PPI-C+A+M/T Seq n/N (%)	PPI-A+Rx n/N (%)	PPI-C+A+M/T Hyb n/N (%)
1st N=60	19/60 (31%)	1/60 (1.6%)	28/60 (46.6%)	2/60 (3.33%)	0/60 (0%)	5/60 (8.3%)	1/60 (1.6%)	0/60 (0%)	1/60 (1.6%)	1/60 (1.6%)	1/60 (1.6%)	0/60 (0%)	1/60 (1.6%)
2nd N=41	4/41 (9.7%)	2/41 (4.8%)	1/41 (2.4%)	23/41 (56%)	0/41 (0%)	1/41 (2.4%)	3/41 (7.3%)	3/41 (7.3%)	4/41 (9.7%)	0/41 (0%)	0/41 (0%)	0/41 (0%)	0/41 (0%)
3rd N=37	5/37 (13.5%)	20/37 (54%)	1/37 (2.7%)	3/37 (8.1%)	0/37 (0%)	1/37 (2.7%)	2/37 (5.4%)	3/37 (8.1%)	0/37 (0%)	0/37 (0%)	2/37 (5.4%)	0/37 (0%)	0/37 (0%)
4th N=32	2/32 (6.2%)	6/32 (18.7%)	0/32 (0%)	0/32 (0%)	13/32 (40.6%)	2/32 (6.2%)	1/32 (3.1%)	0/32 (0%)	0/32 (0%)	3/32 (9.3%)	1/32 (3.1%)	4/32 (12.5%)	0/32 (0%)
5th N=30	23/30 (76.6%)	1/30 (3.3%)	0/30 (0%)	1/30 (3.3%)	2/30 (6.6%)	2/30 (6.6%)	0/30 (0%)	0/ 30 (0%)	0/30 (0%)	1/30 (3.3%)	0/30 (0%)	0/30 (0%)	0/30 (0%)
6th N=7	7/7 (100%)	0/7 (0%)	0/7 (0%)	0/7 (0%)	0/7 (0%)	0/7 (0%)	0/7 (0%)	0/7 (0%)	0/7 (0%)	0/7 (0%)	0/7 (0%)	0/7 (0%)	0/7 (0%)
	60	30	30	29	15	11	7	6	5	5	4	4	4

* Three-in-one single capsule containing bismuth, tetracycline and metronidazole. A, amoxicillin; B, bismuth salts; C, clarithromycin; Conc, concomitant; HDDT, high-dose dual therapy; Hyb, hybrid; L/M, levofloxacin/moxifloxacin; n, number of patients with each treatment regimen; N, number of patients with different line of treatment; M/T, metronidazole/tinidazole; PPI, proton pump inhibitor; Rf, rifabutin; Rx, rifaximin; Seq, sequential; Tc, tetracycline.
